# Drug-Induced Acute Liver Injury Due to Tamoxifen Prophylaxis

**DOI:** 10.7759/cureus.84552

**Published:** 2025-05-21

**Authors:** Rhea Nichani, Sibga Sufian, Azzam A Adhal

**Affiliations:** 1 Obstetrics and Gynecology, Alabama College of Osteopathic Medicine, Dothan, USA; 2 Pediatrics, Alabama College of Osteopathic Medicine, Dothan, USA; 3 Internal Medicine, HCA Florida Gulf Coast Hospital, Panama City, USA

**Keywords:** bilirubin, cholestatic jaundice, drug-induced liver injury (dili), her-2 positive breast cancer, tamoxifen therapy

## Abstract

A 41-year-old female was diagnosed with stage II ductal breast cancer in November 2023. The patient subsequently underwent a bilateral mastectomy and was started on tamoxifen 20 mg as post-operative adjuvant therapy in December 2023. Surveillance positron emission tomography (six months post-mastectomy) confirmed no cancer recurrence prior to symptom onset. Following abdominal pain, nausea and vomiting, pruritus, and jaundice, in the context of an elevated bilirubin level and a 1.5 mm gallstone, tamoxifen treatment was discontinued in July 2024.

In August 2024, the patient presented with recurrent nausea and vomiting, increased abdominal pain, worsening pruritus and jaundice, debilitating back pain, and a weight loss of 20.87 kg. The patient was admitted to the hospital for further workup. Her bilirubin levels were found to be 17.9 mg/dl (normal range: 0.1-1.2 mg/dL). Following subsequent labs and scans such as a hepatobiliary iminodiacetic acid scan, liver biopsy, computerized tomography of the lumbar spine, magnetic resonance imaging of the lumbar spine and bilateral hips, anti-smooth muscle antibody test, and more diagnostic testing, it was determined that the patient was suffering from drug-induced liver injury. After the initiation of steroids and ursodiol, her bilirubin measurements decreased. The patient reported resolution of symptoms a month later, as her bilirubin levels had declined from 17.9 to 2.3 mg/dL over four weeks. This unique presentation of tamoxifen-induced acute liver injury is reversible, and patients on tamoxifen need a frequent comprehensive metabolic panel to detect possible liver injury preemptively.

## Introduction

Adjuvant estrogen-receptor-positive breast cancer treatment and prevention utilize tamoxifen, a nonsteroidal selective estrogen receptor modulator [1]. Tamoxifen has been used for breast cancer since the 1970s and is proven to be efficacious in prolonging survival with good tolerability and few side effects [1]. It does so through competitive inhibition of estrogen, inhibiting DNA and mRNA synthesis, which leads to the inhibition of tumor cell growth [2]. However, long-term use of tamoxifen has been documented to be associated with hepatic dysfunctions, most commonly non-alcoholic fatty liver disease and steatosis [1]. 

The mechanisms leading to certain liver injuries include immunologic reactions, drug metabolism inhibition, changes in bile flow, and dysregulated mitochondrial function [3]. Drug-induced liver injury is any injury to the liver caused by any medication and presents in different subcategories, including hepatocellular, cholestatic, or mixed-type injury [3]. Histological appearance and further laboratory testing assist in identifying an etiology and often drive management. Cholestatic liver injury can be characterized by bile plugging and portal inflammation, elevated serum bilirubin and alkaline phosphatase levels, minimal increases in aminotransferases, and signs of biliary obstruction [4]. Hepatocellular liver injury can have similar findings in terms of inflammation, but can also include necrosis or fibrosis on histologic findings as well as marked increase of transaminases compared to alkaline phosphatase levels [3].

Tamoxifen is the current standard treatment for estrogen receptor-positive breast cancer as it has been proven to reduce the risk of recurrence and mortality of breast cancer [1]. However, rare instances of drug-induced acute liver injury secondary to tamoxifen have been documented, suspected to be a result of CYP2D6-mediated reactive intermediates triggering immune-mediated cholangiocyte injury [2]. Here we report the case of a patient with a history of stage II ductal breast cancer who presented with nausea, vomiting, pruritus, worsening jaundice, weight loss, right upper quadrant pain, and back pain. The diagnosis of cholestatic drug-induced liver injury was independently confirmed by both the liver biopsy and the hepatobiliary iminodiacetic acid scan. The diagnosis informed the change in treatment approach, in terms of discontinuing tamoxifen and starting steroid treatment. Thus, it emphasizes the great value of detecting signs of tamoxifen-induced liver injury as early as possible.

## Case presentation

We present a 41-year-old white female patient with a history of stage II ductal breast cancer, diagnosed in November 2023, status post bilateral mastectomy and chemotherapy, who was started on tamoxifen 20 mg as post-operative adjuvant therapy in December 2023. Surveillance positron emission tomography (six months post-mastectomy) confirmed no cancer recurrence prior to symptom onset. Approximately seven months following initiation of tamoxifen therapy, in July 2024, the patient presented to the emergency room with abdominal pain, nausea and vomiting, pruritus, and jaundice. The evaluation showed a bilirubin of 8 mg/dL and a 1.5 mm stone in the neck of the gallbladder. The patient was hospitalized for a surgical consultation, however, general surgery deferred cholecystectomy due to stable gallstone size (<5 mm), normal common bile duct diameter, and dominant hepatobiliary dysfunction pattern. The symptoms were attributed to possible medication adverse effects, and tamoxifen was subsequently discontinued. One month later, in August 2024, the patient returned with recurrent nausea and vomiting, increased abdominal pain, worsening pruritus and jaundice, debilitating back pain, and a weight loss of 20.87 kg. The patient was admitted and underwent a workup for further evaluation.

Physical examination revealed a blood pressure of 98/67 mmHg, a temperature of 37.4 °C, a pulse of 84 beats/min, and a respiratory rate of 12 breaths/min on room air. The patient was alert and oriented to time, place, and person. Upon general examination, her skin appeared severely jaundiced. Cardiac examination revealed a regular rhythm and rate. Respiratory examination revealed clear lungs bilaterally. Abdominal examination revealed tender hepatomegaly (4 cm below the right costal margin; firm consistency) without splenomegaly or ascites. Lastly, she appeared to have an antalgic gait. 

As depicted in Table 1, bilirubin, alkaline phosphatase, and aspartate aminotransferase were elevated to 17.9 mg/dL, 250 units/L, and 79 units/L, respectively, suggesting hepatic or gallstone etiology. These initial laboratory results prompted further investigation with liver biopsy. Additionally, the patient's thyroid-stimulating hormone and thyroxine levels were low at 0.08 and 2.14, respectively, which is thought to be indicative of central hypothyroidism due to pituitary or hypothalamic dysfunction, which can be caused by drug therapies such as tamoxifen. 

**Table 1:** Relevant laboratory investigations

Laboratory Investigation	Result	Reference Range
Bilirubin	17.9 mg/dL	0.1 - 1.2 mg/dL (Total)
Thyroid Stimulating Hormone (TSH)	0.08 mIU/L	0.4 - 4.0 mIU/L
Thyroxine (T4)	2.14 µg/dL	4.5 - 12.0 µg/dL (Total)
Aspartate Aminotransferase (AST)	79.0 units/L	10.0 - 40.0 units/L
Alanine Transaminase (ALT)	44.0 units/L	7.0 - 56.0 units/L
Alkaline Phosphatase (ALP)	250.0 units/L	44.0 - 147.0 units/L
Ammonia	44.0 µmol/L	15.0 - 45.0 µmol/L
Hemoglobin	10.0 g/dL	12.1 - 15.1 g/dL
Hepatitis A, B, C	Negative	Negative (No infection)
Antinuclear Antibody (ANA)	Negative	Negative
Mitochondrial Antibody (AMA)	Negative	Negative
Smooth Muscle Antibody (SMA)	Negative	Negative
Amino Acid Analysis	Normal	Normal (No abnormality)
Gamma-Glutamyl Transferase (GGT)	22.0 units/L	9.0 - 48.0 units/L
Parathyroid Hormone (PTH)	42.0 pg/mL	10.0 - 65.0 pg/mL
Calcium (Ca)	10.0 mg/dL	8.5 - 10.5 mg/dL
Phosphorus	3.2 mg/dL	2.5 - 4.5 mg/dL
Iron (Fe)	102.0 µg/dL	60.0 - 170.0 µg/dL
Total Iron-Binding Capacity (TIBC)	350.0 µg/dL	240.0 - 450.0 µg/dL

**Figure 1:**
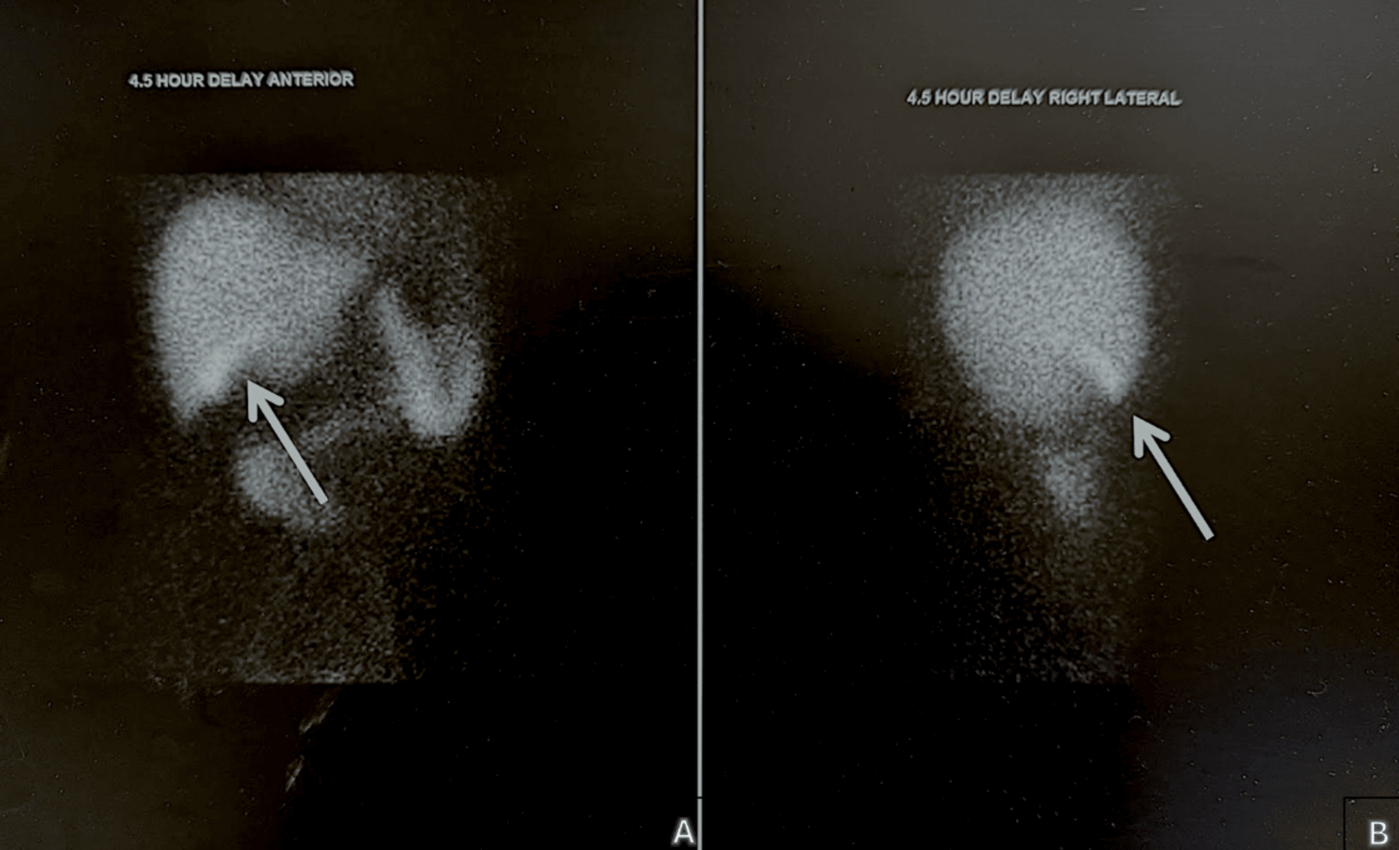
Hepatobiliary iminodiacetic acid scan The above images depict an abnormal scan. Gallbladder activity was not visualized during 60 minutes of sequential imaging. It was eventually visualized at 270 minutes (white arrows) and hence deemed to be consistent with chronic cholecystitis. Anterior view (A) and lateral view (B) are shown above. Additionally, delayed small bowel demonstration may represent partial common bile duct obstruction. Lastly, delayed washout of the radiotracer by the hepatic parenchyma was consistent with hepatocellular dysfunction.

**Figure 2:**
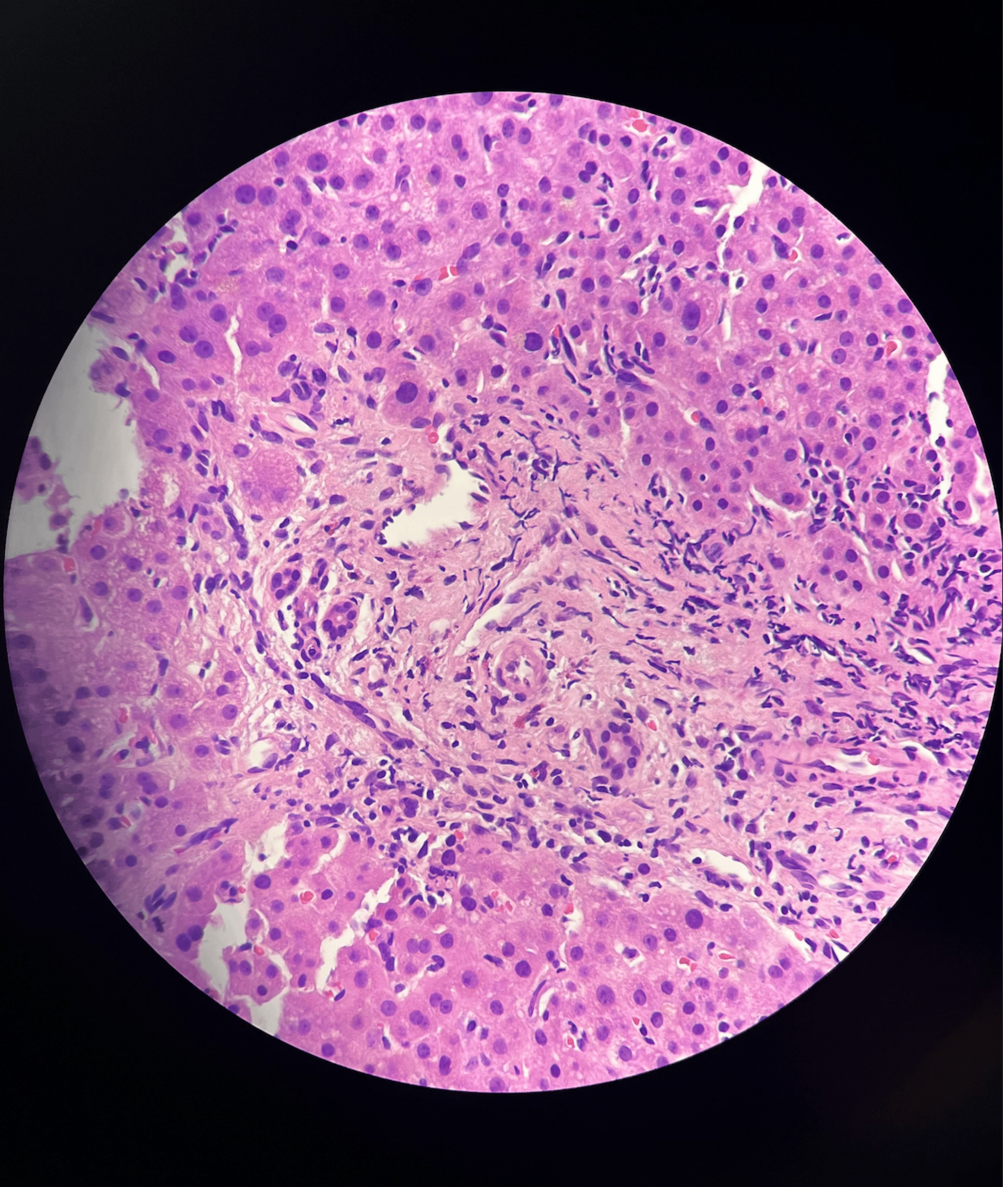
Liver biopsy result of the patient The above figure depicts a liver biopsy of this patient's tamoxifen-induced acute cholestatic liver injury. Cholestatic liver injury, generally clinically characterized by jaundice and pruritus, is accompanied by increases in serum alkaline phosphatase and serum bilirubin [4]. Liver biopsy is generally used to reliably evaluate damage to bile ducts and confirm cholestatic liver injury [4]. This is exemplified by the apparent inflammation with the presence of inflammatory cells, and lobular disarray as shown throughout in this figure.

In terms of imaging investigations, a computerized tomography scan and magnetic resonance imaging scan of the lumbar spine and hips were unremarkable and showed no signs of bony metastasis. A hepatobiliary iminodiacetic acid scan showed delayed gallbladder visualization (270 min) and biliary obstruction (Figure 1). A liver biopsy revealed canalicular cholestasis, portal eosinophils, and hepatocyte dropout further confirming drug induced liver injury (Figure 2).

Based on the above findings, ursodiol 15 mg and prednisone 1 mg/kg were started. The patient was also started on lactulose. Neurology was consulted for pain management, and the patient was started on gabapentin 300 mg. Additionally, the patient began physical therapy for back pain and antalgic gait. Over a four-week period, the bilirubin decreased to 2.3 mg/dl, and the patient was subsequently able to ambulate without difficulty or pain. Additionally, alkaline phosphatase, liver enzymes, thyroid-stimulating hormone, and thyroxine were all monitored over time and normalized as well. After a 29-day hospitalization and an acceptable resolution of symptoms, the patient was discharged home. She continues to follow up with outpatient internal medicine on a quarterly basis.

## Discussion

Our patient experienced a complication of tamoxifen, causing drug-induced liver injury, which presented as cholestatic jaundice with symptoms such as abdominal pain, nausea and vomiting, pruritus, and jaundice. The patient's failure to improve after the initial discontinuation of tamoxifen prompted the need for further evaluation. Drug-induced liver injury is a diagnosis of exclusion based on obtaining a detailed history and physical, hepatobiliary imaging, and a biopsy of the liver [5]. It can mimic many different types of acute and chronic liver conditions, such as autoimmune hepatitis and viral hepatitis [6]. A thorough history and physical are important to establish the temporality of drug initiation and symptoms. However, it may be difficult to establish the diagnosis of drug-induced liver injury as drug-induced liver injury can present with physical manifestations arising within six months after initiation of the drug [5]. Obtaining a liver biochemistry panel can also aid in investigating liver injury and may show elevations in alanine aminotransferases, alkaline phosphatase, and elevated bilirubin [6]. In this case, the diagnosis of drug-induced liver injury was confirmed after excluding autoimmune hepatitis (ANA-), primary biliary cirrhosis (AMA-), and viral hepatitis (serology-), via the Roussel Uclaf Causality Assessment Method (RUCAM) score, which was a probable 8/10. 

Tamoxifen is considered an effective agent in breast cancer prophylaxis but is associated with various adverse effects. The common adverse effects include hot flashes, changes in mood, endometrial proliferation, and venous thrombosis [4]. Recent studies suggested tamoxifen’s effects on the liver most commonly include nonalcoholic fatty liver disease [7]. Adverse effects related to hepatotoxicity are rare, however, the patient’s hepatic changes to tamoxifen were established by the use of a liver biopsy showing cholestatic changes. Symptom onset at seven months aligns with the median tamoxifen-induced liver injury latency (four to nine months) [1], strengthening causality. This is further supported by the resolution of symptoms with the use of corticosteroid therapy and ursodiol treatment, which are reserved for those with cholestatic drug-induced liver injury [8]. Alongside the treatment, the patient’s liver enzymes were followed over time and showed complete resolution. 

Since many cases of drug-induced liver injury are asymptomatic and undocumented, they are often difficult to diagnose. This is especially true in cases that present due to uncommon drug-induced causes, such as in the case of tamoxifen. Even when symptoms are present, they are often nonspecific and may include nausea and vomiting, jaundice, pruritus, right upper quadrant pain, and back pain. Looking only at these symptoms, the differential diagnosis is vast and could encompass other types of hepatic injury, gastrointestinal injury, neurological injury, and more. Hence, it is important that cases of tamoxifen-induced liver injury are documented. Increasing awareness will allow physicians to remain vigilant in conducting the appropriate labs and scans so as to mitigate severe presentations of this disease.

## Conclusions

Long-term use of tamoxifen has been documented to be associated with various hepatic dysfunctions such as fatty liver, steatohepatitis, cirrhosis, and rare instances of acute liver injury. The laboratory results, hepatobiliary iminodiacetic acid scan, confirmatory liver biopsy, as well as the onset of symptomatic liver injury following the initiation of tamoxifen, strongly support the conclusion that tamoxifen is responsible for this patient's acute cholestatic liver injury. A high index of suspicion is required for early diagnosis and treatment of drug-induced liver injury in patients on tamoxifen to prevent complications related to liver injury. Hence, it is important to monitor liver function tests at baseline as well as at one, three, and six months post-tamoxifen initiation. It is also important to monitor for symptoms such as abdominal pain, nausea and vomiting, pruritus, and jaundice. CYP2D6 testing may stratify risk as well. Additionally, because drug-induced liver injury can be caused by various drugs and herbal compounds and can affect various body systems, it is important that we keep this in mind when administering potentially hepatotoxic substances. Overall, although drug-induced liver injury can be detected through diagnosis of exclusion as well as a liver biopsy, there is no clear-cut method of substance indication and diagnosis for this increasingly common condition. Therefore, this topic needs to be further researched.
